# Correlation between ribonucleoside-diphosphate reductase and three replication proteins in *Escherichia coli*

**DOI:** 10.1186/1471-2199-11-11

**Published:** 2010-01-26

**Authors:** M Antonia Sánchez-Romero, Felipe Molina, Alfonso Jiménez-Sánchez

**Affiliations:** 1 Departamento de Bioquímica y Biología Molecular y Genética, Universidad de Extremadura, E06080, Badajoz, Spain

## Abstract

**Background:**

There has long been evidence supporting the idea that RNR and the dNTP-synthesizing complex must be closely linked to the replication complex or replisome. We contributed to this body of evidence in proposing the hypothesis of the replication hyperstructure. A recently published work called this postulate into question, reporting that NrdB is evenly distributed throughout the cytoplasm. Consequently we were interested in the localization of RNR protein and its relationship with other replication proteins.

**Results:**

We tagged NrdB protein with 3×FLAG epitope and detected its subcellular location by immunofluorescence microscopy. We found that this protein is located in nucleoid-associated clusters, that the number of foci correlates with the number of replication forks at any cell age, and that after the replication process ends the number of cells containing NrdB foci decreases.

We show that the number of NrdB foci is very similar to the number of SeqA, DnaB, and DnaX foci, both in the whole culture and in different cell cycle periods. In addition, interfoci distances between NrdB and three replication proteins are similar to the distances between two replication protein foci.

**Conclusions:**

NrdB is present in nucleoid-associated clusters during the replication period. These clusters disappear after replication ends. The number of these clusters is closely related to the number of replication forks and the number of three replication protein clusters in any cell cycle period. Therefore we conclude that NrdB protein, and most likely RNR protein, is closely linked to the replication proteins or replisome at the replication fork. These results clearly support the replication hyperstructure model.

## Background

Initiation of chromosome replication is a key and tightly regulated event in the *E. coli *cell cycle. Initiation starts by the binding of DnaA protein to the *oriC *sequence. This facilitates DNA strand opening, and allows the subsequent loading of the primosomal proteins DnaB helicase and DnaG primase, and, finally, the DNA polymerase III holoenzyme, forming the replisomes that will lead to bidirectional chromosome replication [1]. DNA synthesis requires a balanced supply of the four dNTPs in all living cells. NDP reductase (RNR) is an essential enzyme for the synthesis of these precursors in most organisms [2,3]. RNR is a tetramer made of two homodimers - subunit R1 coded by the *nrdA *gene and subunit R2 coded by the *nrdB *gene [2]. A body of evidence supports the idea that RNR and the dNTP-synthesizing complex must be closely linked to the replication complex or replisome [4]. The dNTP concentrations required for optimal *in vivo *DNA synthesis appear to be four times higher than measured cellular pools [4,5]. Furthermore, allosteric regulation of RNR activity, controlled by the ATP to dATP ratio, will not function in free cytoplasmic enzymes where the ATP pool is 20 times greater than dATP [6], so that this regulation can only be achieved in a highly concentrated dATP environment not present in whole cells [7]. Close proximity of the dNTP-synthesizing complex to the replisome inside an enclosed structure would provide the required precursors for DNA synthesis in the place where they are needed and at the required concentrations. Other observations supporting the suggested compartmentation of precursors show that permeabilized *E. coli *cells incorporate dNMPs into DNA more efficiently than dNTPs, and inhibition of nucleoside diphosphate kinase inhibits incorporation of both dNMPs and dNTPs [8]. Furthermore, a direct interaction of RNR with protein MreB, involved in chromosome segregation, and with protein DnaN, the β-clamp subunit of DNA polymerase III, have been described by Butland *et al. *[9]. Interactions between dNTP synthesis and proteins involved in DNA replication have also been shown in T4 [8,10-12] and in eukaryotic cells [13,14].

In a previous paper [15] we showed that the thermosensitive RNR protein coded by allele *nrdA101*, which loses its activity *in vitro *after a few seconds [16], *in vivo *maintains its activity for more than 160 min. Bacterial strains with this mutant allele perform replication fork reversal (RFR), as thermosensitive replication proteins do at the restrictive temperature, unrelated to the inhibition of deoxynucleotide synthesis [17]. Furthermore, the growth of these mutant cells at the non-permissive temperature also affects nucleoid organization and chromosome segregation in a manner independent of the protein's enzymatic activity [18], which might be explained by the physical interaction of RNR with protein MreB [9]. These results point towards the requirement of a precise structure of RNR, in addition to its reductase activity, to maintain an active replication fork and to allow chromosome segregation and a precise cell division [18]. Together, this evidence suggests the presence of a replication hyperstructure where all the functions required for precursor biosynthesis and chromosome replication and segregation would be assembled in a higher order structure [15,19,20].

This hyperstructure should require RNR, and consequently the dNTP-synthesizing complex, to be associated with replication forks. It has been suggested that this replication hyperstructure is disrupted when the supply of nucleotides is limited or when thymidylate synthetase is mutated [21]. Recently, RNR has been suggested to be a central element of this hyperstructure [22]. However, a later study indicated that RNR may be evenly distributed throughout the cell since no discrete foci were observed [23]. Consequently, we were interested in establishing whether RNR is found in discrete foci or is dispersed throughout the cell, and, if present, whether those foci were in close proximity to the replication complex.

Using fluorescence microscopy and epitope tagged proteins, we determined the location of RNR and three replication proteins in slowly growing cultures of *E. coli*. We observed discrete foci formation of the NrdB::3×FLAG protein. When this tagged protein was analyzed together with immunolabeled SeqA protein, which binds newly replicated hemimethylated DNA and consequently defines the localization of replication forks [24], with DnaB helicase, or with DnaX τ subunit, we observed similar average numbers of foci of the four tagged proteins in the full bacterial population, and we also found similar numbers of the four foci when cells were grouped according to the number of predicted replication forks. Furthermore, inter-foci distances between NrdB and each of the three selected replication proteins were similar to or even less than those between replication proteins. These results support a coordinated organization of NrdB protein, and consequently RNR protein, with other replication proteins, and provide support for the replication hyperstructure model.

## Results

### NrdB is found in discrete foci that correlate with the number of replication forks

A recently published work showed that immunolabeled RNR does not form discrete foci in *E. coli *suggesting that this protein might be dispersed throughout the cell [23]. This result called into question our interpretation of the results of our previous studies as indicating a close association of RNR with the replisome [15,17,18]. Consequently, we were interested in confirming or rejecting this interpretation of our data by analyzing the capability of immunolabeled 3×FLAG-tagged RNR to form foci.

**Figure 1:**
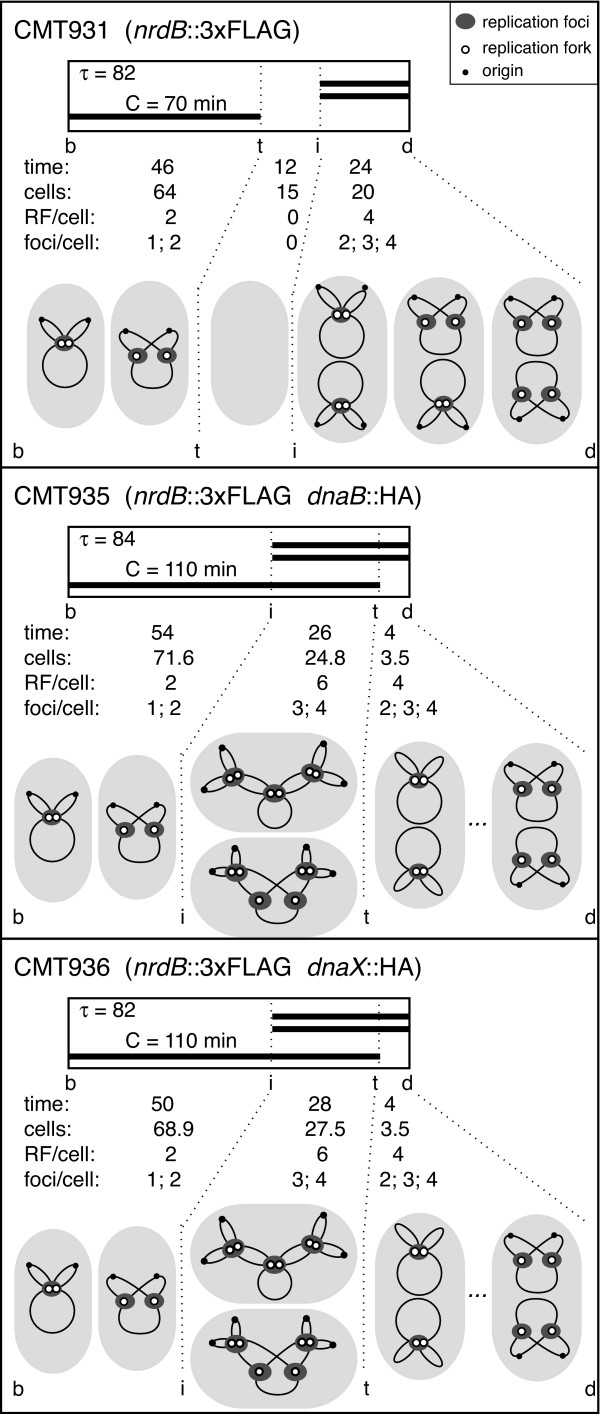
**Cell cycle events and chromosome structure**. Cell cycle events and chromosome shapes relative to cell age in strains CMT931, CMT935, and CMT936 growing in glycerol minimal medium at 37°C. τ is the doubling time in minutes. *C *is the time in minutes required to replicate the chromosome. Thick bars represent replicating chromosomes. *Time *refers to the length in minutes of each of the three periods determined by cell birth (*b*), initiation of replication (*i*), termination of replication (*t*), and cell division (*d*). *cells *is the frequency of cells, in percentages, in each of the three periods. *RF/cell *is the number of replication forks per cell, and *foci/cell *is the expected number of foci per cell of a fluorescent replication protein depending on the association state of the sister replication forks, in each of the three cell cycle periods. The inset shows the symbols for the origin of replication (small black circle), for each replication fork (open circle), and for the foci of an immunostained replication protein (grey oval).

Analysis of slowly growing *E. coli *cells greatly facilitates interpretation of replication data due to reduced overlap of consecutive cell cycles, and the lower number of replication forks per cell. Faster growth rates cause increasing numbers of overlapping replication cycles, and consequently more replication forks per cell. Furthermore, two or more replication forks can aggregate and their co-localization correlates with the overlapping of replication cycles, and therefore the discrepancy between the number of forks and foci per cell increases with the growth rate in wild-type cells [25-27]. In the present work cells were grown in glycerol minimal medium at 37°C and cell cycle parameters were studied by flow cytometry (data not shown) as described in Methods. Insertion of the 3×FLAG epitope at the C terminus of NrdB did not change the cell cycle parameters from those of the parental strain (Figure 1), which suggests that the tagged RNR protein has no detrimental effect upon bacterial growth and chromosome replication. Insertion of the HA epitope at the C terminus of DnaB helicase or τ subunit DNA polymerase, however, increased the replication period to 110 min and cut the *D *period down to 4 min in both strains. These results make it possible to obtain a complete description of the proportion of cells with their expected chromosome structure and the number of replication forks. The expected number of foci for any replication protein was deduced by considering the association of the two sister replication machineries as described elsewhere [26] (Figure 1).

**Figure 2:**
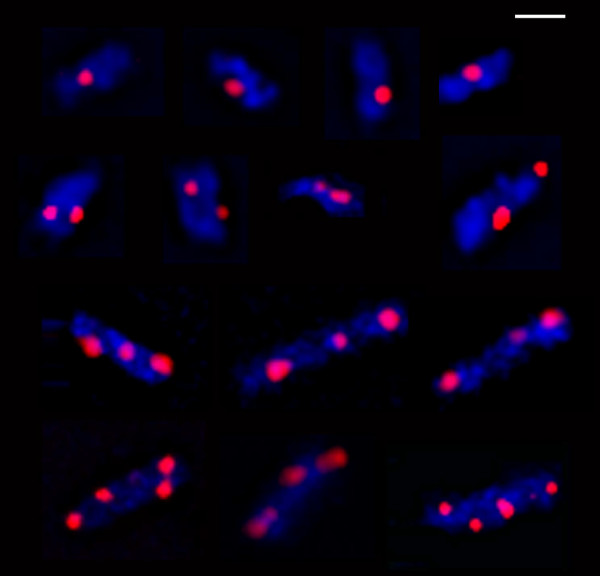
**Fluorescence microscopy of tagged-NrdB cells**. Fluorescence microscopy images of cells of CMT931 (*nrdB*::3×FLAG) immunolabeled with Cy3 conjugated anti-FLAG antibody (red) and stained with Hoechst 33258 (blue). The bar represents 1 μm.

Fluorescence microscopy of CMT931 immunolabeled with Cy3 conjugated anti-FLAG antibody showed that all cells contained from one to four discrete foci (Figure 2), and no diffuse fluorescence could be seen in any cell. Cells were also stained with Hoechst 33258 to detect bacterial nucleoids. The micrographs obtained showed that all NrdB foci were localized within the nucleoid boundary or in its periphery. Consequently we can conclude that RNR is essentially concentrated in discrete clusters and not spread throughout the cell, and that these clusters are associated with the nucleoid.

**Figure 3:**
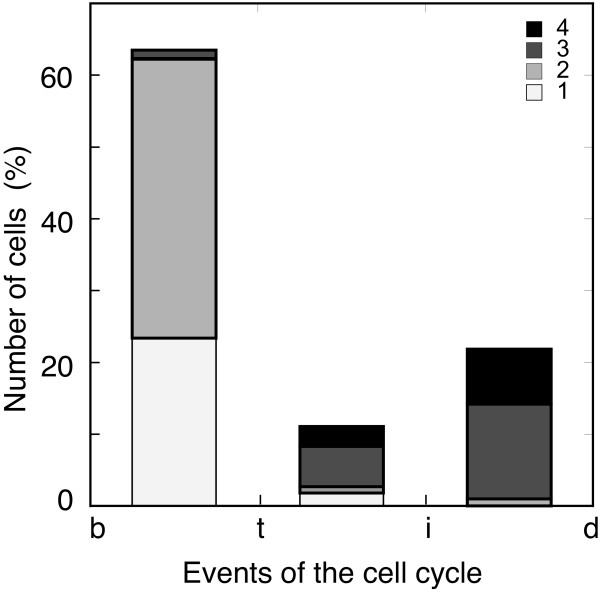
**NrdB foci and replication forks**. Percentage of cells containing different numbers of NrdB::3×FLAG foci per cell of strain CMT931, distributed into the three periods of the cell cycle defined as from birth (*b*) to termination of replication (*t*), from termination to initiation (*i*), and from initiation to cell division (*d*), as described in Figure 1. Number of cells analyzed was 426. The inset shows the colour code for the four numbers of foci per cell.

A higher level of information can be achieved by comparing the number of foci per cell with the number of replication forks in each period of the cell cycle. Given that cell length increases exponentially throughout the cell cycle, and notwithstanding the variation in length at birth and at division [28,29], it can be used to estimate the age of a cell. We therefore used cell length to sort the bacteria into the three periods determined by the initiation and termination of chromosome replication. In each group of cells, the number of NrdB foci corresponded very closely to what was predicted for the number of replication forks (Figure 3). This high level of equivalence between NrdB foci and the replication forks suggests a close relationship between the RNR and the replisome.

**Figure 4:**
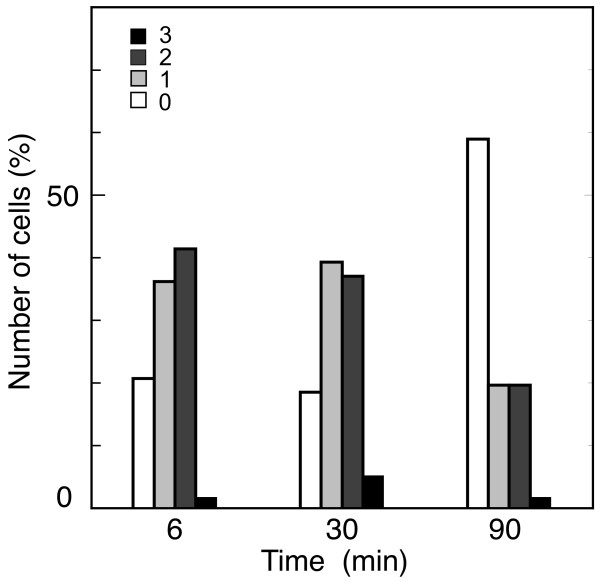
**Analysis of NrdB foci during synchronous replication**. Number of NrdB foci in a CMT934 (*nrdB*::×FLAG *dnaC2*) culture after the three-step temperature shifts for synchronous initiation of one round of chromosome replication. Samples were withdrawn at 6, 30, and 90 min after the beginning of the 6 min period at 30°C to allow one initiation round. Number of cells analyzed was 261. The inset shows the colour code for the number of foci per cell.

A hypothesized connection between RNR and the replication fork would predict that NrdB foci should decrease after replication ends. In order to investigate this issue, we used a synchronized culture for initiation of chromosome replication of strain CMT934 which includes allele *dnaC2 *together with *nrdB*::3×FLAG. Fluorescence microscopy of these synchronized cells showed that, when they were replicating, 80 per cent of cells had one or two NrdB foci (Figure 4). The 20 per cent without foci could denote cells that did not initiate replication during the activation of the replication period. After replication ended, the number of cells without NrdB foci increased threefold, and the number of cells containing NrdB foci decreased. These results show that, once replication forks are expected to have finished [30], the number of NrdB foci, and consequently RNR assembly, also diminishes, thus supporting the proposed connection between the RNR and the replication fork.

### NrdB foci are found in essentially the same numbers as foci from SeqA clusters, DnaB helicase, and DNA polymerase III τ subunit

We then were interested in comparing the average number of NrdB foci in an exponentially growing culture with that of three proteins associated with replication forks: SeqA protein, known to bind newly replicated DNA and, therefore, the replication fork [24], and two interacting components of the replisome - DnaB helicase and the DNA polymerase III τ subunit coded by the *dnaX *gene. For this purpose, we constructed strain CMT935, containing a C-terminal HA-tagged DnaB helicase and the NrdB::3×FLAG protein, and strain CMT936, containing a C-terminal HA-tagged DnaX subunit and the NrdB::3×FLAG protein.

**Figure 5:**
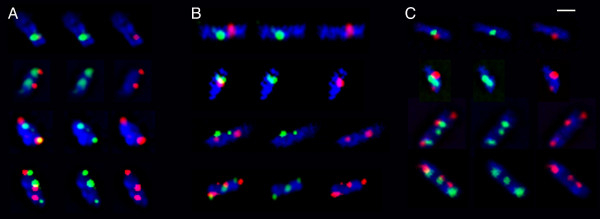
**Fluorescence microscopy of tagged-NrdB and SeqA, or DnaB, or DnaX cells**. Fluorescence microscopy images of cells of CMT935 (*nrdB*::3×FLAG *dnaB*::HA) (A) or CMT936 (*nrdB*::3×FLAG *dnaX*::HA) (B) immunolabeled with Cy3-anti-FLAG (red) and FITC-anti-HA (green), or CMT931 (*nrdB*::3×FLAG) immunolabeled with Cy3 conjugated anti-FLAG (red) and FITC-anti-SeqA (green) antibodies (C). Cells were also stained with Hoechst 33258 for nucleoid visualization. Each group of cells shows nucleoid (blue) and, from left to right: both green and red, only green, and only red fluorescence. The bar represents 1 μm.

**Figure 6:**
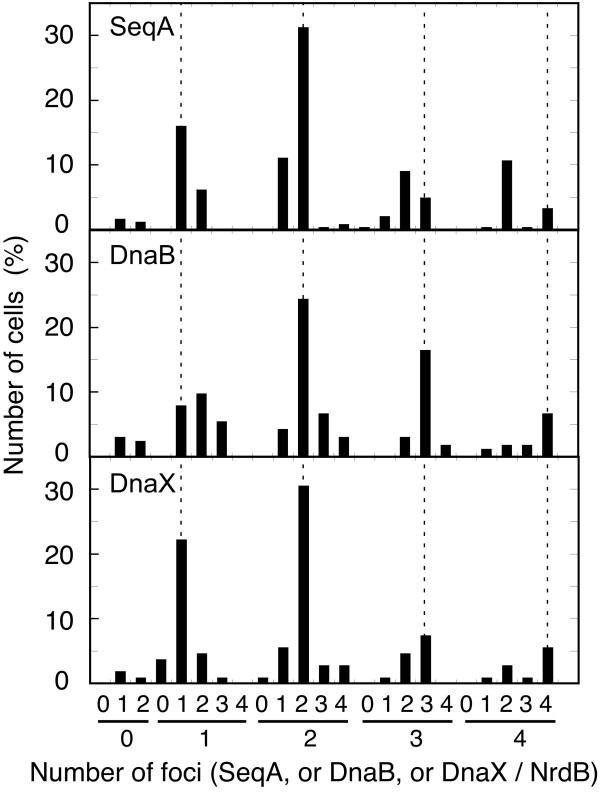
**NrdB foci related to SeqA, DnaB, and DnaX foci**. Frequency of the number of foci of NrdB together with SeqA fluorescent proteins in strain CMT931 (*nrdB*::3×FLAG), or with DnaB helicase in strain CMT935 (*nrdB*::3×FLAG *dnaB*::HA), or with DNA polymerase III τ subunit in strain CMT936 (*nrdB*::3×FLAG *dnaX*::HA) in mid log cells growing in glycerol minimal medium at 37°C. Numbers in the upper position of the horizontal axis are the number of foci of any of the three replication proteins present in a cell containing the number of NrdB foci indicated by the number in the lower position. Dotted lines identify cells with the same number of both foci. Numbers of cells analyzed were 129, 172, and 110, respectively.

Immunolabeling of RNR together with any of the aforementioned proteins revealed the presence of foci for every protein (Figure 5). Counts of the numbers of each kind of foci per cell gave a clear-cut pattern of similarity between NrdB foci and those of the replication proteins (Figure 6). All four proteins were represented in the culture by 1, 2, 3, and 4 foci, with 1 and 2 foci being predominant in consonance with the number of predicted foci (Figure 1). Furthermore, the number of foci per cell of every replication protein was very similar to the number of NrdB foci. From these results it seems to be evident that the RNR protein is closely related to the replication proteins, and suggests that it should be mainly concentrated at the replication forks.

It is worth noting that those cells containing 2, 3, or 4 NrdB foci, which have a high consistency with the number of DnaB and DnaX foci, were found to have the same number or more of those foci than of SeqA foci (Figure 6A). The number of DnaB helicase and DnaX τ subunit foci, also greater than that of SeqA foci, could be explained by pre-replication assembly of the replisome proteins [31]. Likewise, the greater number of NrdB foci relative to those of SeqA can be explained by assuming that this protein was also pre-assembled together with other replisome proteins before replication initiated.

### The number of NrdB foci throughout the cell cycle matches with those of SeqA, DnaB helicase, and τ subunit

**Figure 7:**
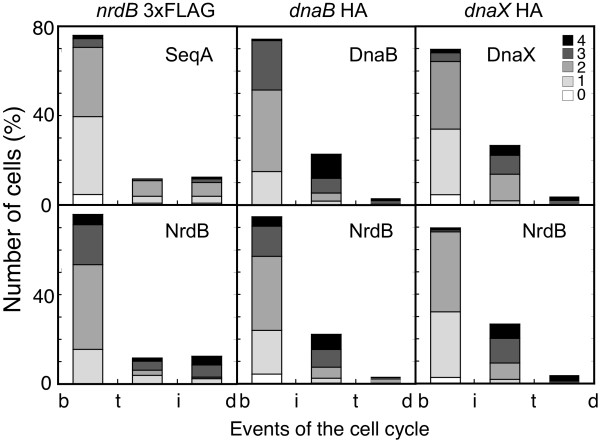
**NrdB foci and replication proteins foci along the cell cycle**. Percentage of cells containing different numbers of foci during the three cell cycle periods described in Figure 1. Foci of fluorescent proteins were from SeqA and NrdB in strain CMT931 (*nrdB*::3×FLAG); DnaB helicase and NrdB in strain CMT935 (*nrdB*::3×FLAG *dnaB*::HA); and DnaX τ subunit and NrdB in strain CMT936 (*nrdB*::3×FLAG *dnaX*::HA). Numbers of cells analyzed were 129, 172, and 110, respectively. The inset shows the colour code for the number of foci per cell.

To analyze the foci distribution during the cell cycle, we arranged the cells into the three cycle periods determined by the initiation and termination of chromosome replication. For each group of cells, the numbers of foci of each of the four tagged proteins were determined. The data displayed in Figure 7 reveal very strong agreement between the number of NrdB foci (Figure 7, lower panels) and the numbers of foci of each replication protein (Figure 7, upper panels) in each cell cycle period. These results provide further evidence for the strong correlation between the RNR and the replication proteins.

There was only a slight inconsistency observed in the relationship between NrdB and SeqA foci with there being more of the former than of the latter. As mentioned in the previous section, this inconsistency could be ascribed to pre-replication assembly of the replication proteins.

### NrdB foci localize near SeqA, DnaB helicase, and τ subunit foci

Notwithstanding the coincidence between the number of NrdB foci and replication protein foci being a good indication of the association between RNR and the replisome proteins, a further property of this relationship should be the close subcellular location of the associated proteins. The possible nearness of RNR to the replication proteins was determined by measuring the distance between the pairs of foci. In particular, we measured inter-foci distances between NrdB foci and SeqA, or DnaB helicase, or DnaX τ subunit foci in cells containing the same number of both tagged proteins to obviate pre-assembly generated foci. For a correct understanding of the observed dimensions, and as a control of closeness between proteins known to be at the replication fork, we also measured distances between SeqA and DnaB helicase foci and between SeqA and τ subunit foci. The observed inter-foci distances between NrdB and each of the replication proteins gave very similar values to those found between SeqA and DnaB or DnaX foci (Table 1). Interfoci distances were measured from the centres of foci. As foci have an average diameter of 0.26 μm, one can deduce from the data in Table 1 an overlap of foci in 90, 52, and 80 per cent of foci pairs of NrdB with SeqA, DnaB, and DnaX, respectively. From the same table one can deduce that DnaB and DnaX protein foci, known to be at the replication fork, overlap with SeqA foci in 73 and 78 per cent of the studied pairs of foci, respectively.

**Table 1:** Mean inter-foci distances (μm) between tagged replication proteins

Proteins	Mean	St. Dev.	Number of foci-pairs measured
NrdB SeqA	0.13	0.10	82
NrdB DnaB	0.25	0.22	217
NrdB DnaX	0.15	0.13	141
SeqA DnaB	0.16	0.16	65
SeqA DnaX	0.15	0.14	68

**Figure 8:**
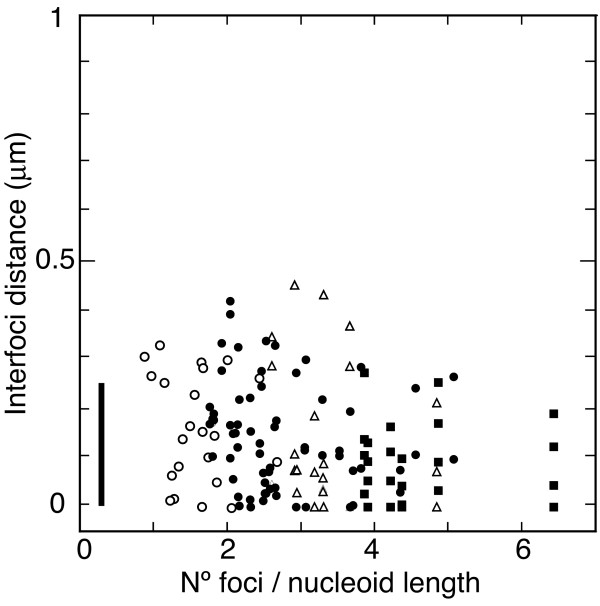
**Distances between NrdB and DnaX foci relative to foci per cell**. Interfoci distances between NrdB and DnaX τ subunit relative to foci compaction in cells containing 1 (open circles), 2 (solid circles), 3 (open triangles), or 4 (squares) pairs of foci. Foci compaction is expressed by the number of pairs of foci per nucleoid length. Vertical bar represents the focus diameter. Number of cells analyzed was 110.

To rule out the a priori not unlikely possibility of random localization, we studied inter-foci distances between NrdB and replication protein foci relative to the number of pairs of foci per nucleoid length. The rationale of this approach was that, with a random distribution of foci, the higher the number of foci per cell length the smaller the inter-foci distances. Inter-foci distances between NrdB and DnaX τ subunit in cells with different numbers of foci per nucleoid length are shown in Figure 8. The data show that distances between NrdB and τ foci are independent of foci compaction, and hence can not be explained by a random distribution. Very similar results were obtained when this analysis was performed with NrdB and DnaB foci, or with NrdB and SeqA foci (data not shown). Consequently, these results establish the high degree of nearness of the proteins studied, and confirm that RNR protein can not be farther away from the replisome than are other replication proteins.

## Discussion

According to Mathews' and our own work, we had proposed that a replication hyperstructure should be the association of all the proteins required for replication, i.e., the replisome together with the dNTP-synthesizing complex and most likely with proteins required for chromosome segregation, all included in some membrane structure [4,15,18-20]. This model would require RNR protein to be localized very close to the replication proteins. This should cause the fluorescence of immunolabeled RNR protein to be concentrated in discrete foci, and these foci should also be present in similar numbers to other fluorescent replication protein foci and in close proximity to them.

A recent work had put the likelihood of this model into doubt by showing that immunolabeled RNR did not appear in foci but seemed to be dispersed throughout the cell [23]. We were therefore interested in a further study to confirm or invalidate this result and in consequence our model.

We first decided to label RNR protein by tagging a short epitope to the C-terminus of R2 subunit as an alternative to the more severe binding of a fluorescent protein, which would disturb a likely higher structure. The correct functionality of this tagged protein was verified by checking that growth rate and replication parameters were the same as the parental strain. When the strain CMT931, containing the NrdB::3×FLAG tagged polypeptide, was immunolabeled with a specific anti-FLAG antibody and observed by fluorescence microscopy, all cells were perceived to bear from one to four discrete foci. The difference with the findings in the work of Watt's group might be ascribable to the epitope used to tag the protein [23]. In that work they used a derivative of GFP to tag NrdB. The RFP or GFP (≈ 34 kDa) fusion can compromise the full function of the target protein that can change the pattern of its localization [32]. This possible alteration is more crucial when a higher order structure is being proposed for RNR.

Cell size was also measured, and cells were distributed by their length into the three periods of a cell cycle, determined by the initiation and termination of replication, in order to study the relationship between predicted fork numbers in each period and the observed foci number. However, there are two factors that could affect this assignment strategy. On the one hand, the distributions of lengths at birth and at division are known to have coefficients of variation of 13.5 per cent [28], and consequently so do the cell sizes at any given cell age. And on the other, replication proteins can be assembled into a pre-replication structure that would lead to more foci than replicating forks [31]. Both effects could explain the number of NrdB foci found in the period between termination and initiation shown in Figure 3 where no replication forks are expected. Nevertheless, the good agreement found between the number of immunolabeled fluorescent NrdB foci per cell cycle period and the number predicted for replication protein foci provides clear support to sustain the association of the RNR with the replication fork.

A prediction of this association would be that NrdB foci should decrease after replication ends. The presence of NrdB foci during the replication time and their decline after termination of replication was verified by fluorescence microscopy of a culture synchronized for initiation of chromosome replication. The treatment used provides reasonable synchronization of one chromosome replication round, but not all cells initiate replication. Moreover, after replication should have terminated, a number of cells were still replicating, most likely due to a low elongation rate of the *dnaC2 *mutant strain at the high temperature [30]. This drawback prevents the disappearance of all replication forks, and could be the source of cells with NrdB foci after 90 min at the high temperature. A fraction of this number of non-replicating cells containing NrdB foci could also be due to the aforementioned pre-assembly of the replication complexes.

In order to ascertain that RNR protein was indeed associated with the replication fork, we investigated the number of fluorescence immunolabeled NrdB foci together with foci from a C-terminal HA-tagged DnaB helicase, or from a C-terminal HA-tagged DnaX τ subunit from the DNA polymerase holoenzyme, or from immunolabeled fluorescent SeqA. Both measurements of foci per cell in the whole culture and per individual cell cycle period in the three strains gave very similar values for any of all three replication protein foci as well as for NrdB foci (Figures 6 and 7).

The greater number of DnaB helicase and DnaX τ subunit foci relative to those of SeqA could be explained by the pre-replication assembly of the replisome proteins [31]. Likewise, the greater number of NrdB foci relative to those of SeqA can be explained by assuming that this protein was also pre-assembled together with the replisome proteins before replication initiated. This might indicate that the whole replication hyperstructure could be organized some time before replication is initiated. This explanation could account for the number of CMT931 cells found without SeqA foci but with NrdB foci.

An implication of the association between RNR and the replisome would be the nearness of RNR to any replisome protein. We determined the distance between NrdB foci and those of SeqA, DnaB helicase, and DnaX τ subunit. The data showed that almost all distances found between NrdB foci and any other replication protein foci were the same as the distances between DnaB or τ foci and SeqA foci. Furthermore, our data show that these distances are not due to random localization corresponding to foci compaction. Therefore, we can conclude that NrdB protein is localized near the replication fork.

The above data lead to three important conclusions: (*i*) NrdB, and consequently RNR, is located in discrete foci. (*ii*) The number of NrdB foci per cell is the same as the predicted number of replication forks, and it is the same as the number of foci of the replisome proteins DnaB helicase and DNA polymerase III τ subunit. (*iii*) The localization of NrdB foci is not random; instead they are close to SeqA, DnaB, and DnaX τ subunit. With these results, the only convincing explanation is that RNR protein is associated with the replication complex. This association would predict that other proteins from the dNTP synthesizing complex could also be related to the replication fork, most likely in a higher order structure, the replication hyperstructure. High local concentrations of molecules can be attained by co-localization of proteins, and new functions can emerge [33]. Such organization in a replication hyperstructure might permit channeling and the compartmentation of precursors so as to provide a balanced supply of the four dNTPs at the concentration required for high speed processive chromosome replication.

## Conclusions

NrdB protein, and most likely RNR protein, is assembled in clusters associated with nucleoids. The observations that (*i*) the number of NrdB foci correlates with the number of replication forks in any cell cycle period, (*ii*) the end of replication causes the disappearance of most of the NrdB foci, (*iii*) the number of NrdB foci is very similar to that of SeqA, DnaB, and DnaX foci in any cell cycle period, and (*iv*) interfoci distances between NrdB and the three replication protein foci are very similar to those between replication proteins, allow us to conclude that NrdB clusters emerge and are related to the replisome during the bacterial chromosome replication period. These results provide clear support for the replication hyperstructure hypothesis.

## Methods

### Bacterial strains, plasmids and growth conditions

All strains used were derived from *E. coli *K-12 (Table 2). CMT931, a CM735 containing 3×FLAG-tagged *nrdB *gene, was used to immunolocalize the R2 subunit of RNR. Strains CMT935 and CMT936, containing HA-tagged *dnaB *gene and HA-tagged *dnaX *gene, respectively, and 3×FLAG-tagged *nrdB *gene, were used for co-localization of DnaB helicase and subunit τ of DNA polymerase holoenzyme and RNR protein. For their construction, strain CMT927, a pKD46-harbouring CM735, was used. Strain EBO193 is CM735 Δ*seqA *and was used for nonspecific absorption of antibody anti-SeqA. Bacteria were incubated at 37°C in M9 minimal glycerol medium supplemented with requirements. When required, kanamycin was used at 25 μg ml^-1^. Strain CMT934 (*dnaC2 nrdB*::3×FLAG) was used for immunolocalization of RNR in synchronized cells.

**Table 2:** *Escherichia coli *K-12 strains used in this work

Strain	Genotype	Source
CM735	metE46 trp3 his4 thi1 galK2 lacY1 mtl1 ara9 tsx3 ton1 rps8 supE44 l-	Dr K. Skarstad
CMT927	CM735/pKD46	This work
CMT931	CMT927 *nrdB*::3×FLAG	This work
CMT934	CMT931 *dnaC2 thr*::Tn10	This work
CMT935	CMT931 *dnaB*::HA	This work
CMT936	CMT931 *dnaX*::HA	This work
EBO193	CM735 Δ*seqA*	Dr K. Skarstad

Plasmid pKD46 is a temperature sensitive replicon expressing λ *red *genes under the arabinose-inducible P*araBAD *promoter [34] and was used for linear DNA transformation. Plasmid pSUB11 and plasmid pSU314 were used as template for the amplification of DNA fragments containing the 3×FLAG and the influenza virus HA epitope sequences, respectively [35]. pSUB11 and pSU314 carry chloramphenicol- and kanamycin-resistance genes, respectively, flanked by the recognition sites (FRT sites) of the yeast FLP recombinase in direct repeats [36].

### Construction of epitope-tagged proteins

3×FLAG epitope was attached to the C-terminal sequence of the R2 subunit of RNR, and HA epitope was tagged to the C-terminus of DnaB helicase and of DNA polymerase III holoenzyme τ subunit, essentially as described [34,35]. Briefly, a DNA molecule containing the epitope-encoding sequence and the kanamycin- or chloramphenicol-resistance gene were amplified by PCR. For protein tagging, primers shown in Table 3 were used. Forward primers contained 36, 39, and 33 bases from downstream of the gene to be tagged and 20 bases (underlined) from the upstream sequence of the epitope-encoding sequence; reverse primers contained 39, 35, and 35 bases from the downstream sequence following the end of the gene to be tagged, and 20 bases (underlined) from the 3'-end of the FRT sequence downstream of the kanamycin or cloramphenicol gene. In this approach genes are not replaced, and 3×FLAG or HA were inserted in wild-type genes through homologous recombination after the last C-terminal amino acid.

**Table 3:** Primers used for protein tagging

Tagged protein	Primer direction	Primer sequence
NrdB	forward	GAAGTGGACACCGACGATTTGAGTAACAACCAGCTCGACTACAAAGACCATGACGG
	reverse	TCAAATTTTTCCAATCGCCACTAATTGTTCCATGCACATCATATGAATATCCTCCTTAG

DnaB	forward	CGCTTCGACAACTATGCGGGGCCGCAGTACGACGACGAATTCTATCCGTATGATGTTCC
	reverse	GTGTTCCTTGATAAGTGTTTGCTTTAATTACCTAATATATGAATATCCTCCTTAG

DnaX	forward	GCGGAGCTGGATGAAGAAAGTATCCGCCCCATTTTCTATCCGTATGATGTTCC
	reverse	GGTTTCTCTCTCAATCACGTTAAGGATGACGAACGTATATGAATATCCTCCTTAG

Underlined bases in forward primers correspond to the upstream of the epitope-encoding sequence.

Underlined bases in reverse primers correspond to the 3'-end of the FRT sequence downstream of the kanamycin or chloramphenicol gene.

Strain CMT927 expressing λ *red *functions was transformed with linear DNA obtained by PCR. A final 1 mM L-arabinose was used for the induction of the promoter. Kanamycin or chloramphenicol resistant transformants were selected in selective plates at 30°C. Bacterial chromosomes containing 3'-terminal-tagged genes were verified by PCR and tagged proteins by western blot using the same antibodies as in the fluorescence microscopy (see below).

### Immunofluorescence microscopy

Cell preparation and staining were performed as described [37]. Samples of 1.5 ml from an exponentially growing culture were withdrawn at 0.15 OD_550_, and cells were collected by centrifugation, washed, resuspended in 1 ml TE buffer, and fixed by adding the same volume of cold 74% ethanol.

Immunostaining of SeqA protein was performed using cell extracts from strain EBO193 for absorption of nonspecific SeqA antibody as described previously [7].

Ethanol-fixed cells (100 μl) were stained with mouse monoclonal anti-FLAG M2-Cy3 antibody (Sigma-Aldrich) to immunolocalize RNR, or with mouse monoclonal anti-HA (clone 7) antibody (Sigma-Aldrich) and a secondary anti-mouse antibody conjugated to Alexa 488 (Molecular Probes) for DnaB helicase and τ subunit localization. When both antibodies were used in the same preparation, anti-FLAG antibody was used as the last treatment and after extensive PBS washing. SeqA was labeled with rabbit polyclonal anti-SeqA antibody and an anti-rabbit secondary antibody conjugated to FITC (Sigma-Aldrich), as previously described [24]. Immunostained cells were stained with Hoechst 33258, 1.5 mg ml^-1^, in 10 μl mounting medium (40% glycerol in 0.02 M phosphate buffered saline, pH 7.5). 20 μl of ethanol-fixed cells was spread onto a poly-L-lysine-coated slide, and dried at room temperatures. Slides of stained samples were stored at room temperature in the dark.

Images were captured with a Delta Vision optical microscope (Applied Precision) equipped with a 100× UPLS Apo objective and filter sets for DAPI, FI-GF-BF, and RD-TR-PE filters, and were deconvolved using 15 iterations of the Delta Vision constrained iterative deconvolution software (Applied Precision). From every field, different micrographs using each of the three filters were taken. The deconvolved images were saved in TIFF format and imported into ImageJ software for cell and nucleoid size measurements and localization of foci centres (Wayne Rasband, Research Services Branch, National Institute of Mental Health, USA).

### Cell cycle parameters

Growth rates of exponentially growing bacteria were determined by the absorbance of the cultures at 550 nm. For the cell cycle parameter measurements, cells were treated with rifampicin, 150 μg ml^-1^, and cephalexin, 50 μg ml^-1^, to inhibit initiation of replication and cell division, respectively. After three hours of treatment, cells were fixed, and the number of chromosomes per cell was measured by flow cytometry using a FAC Star DIVA (Becton Dickinson) instrument, essentially as previously described [37]. The time required for chromosome replication, *C*, and the time from the termination of replication to cell division, *D*, were determined from the number of cells before and after the initiation of replication in the initiation time equations [38,39].

Briefly, *C *can be obtained from the average amount of DNA per cell obtained by cytometry after the rifampicin and cephalexin treatment relative to that amount before the treatment (*dG*). This relative increment in the amount of DNA per cell is: *dG *= (2^*n *^*n *ln2)/(2^*n *^- 1), and *n *= *C*/τ [40]. If we designate *R *= (*C*+*D*)/τ and *r *being the integer value of *R*, analysis of bacterial cell cycles with any possible overlapping results in a number of origins per cell before and after initiation of replication equal to 2^*r *^and 2^(*r*+1) ^respectively. These number of origins correspond to the number of full replicated chromosomes per cell at the end of the rifampicin and cephalexin treatment. The time at wich replication initiates, *t*_*i*_, can be deduced to be *t*_*i *_= 1 + *r *- *R*. The frequency of cells before and after the initiation of replication can be deduced from Powell's age distribution [38] to be 2 - 2^(*R*-*r*) ^and 2^(*R*-*r*) ^- 1 respectively, and are obtained experimentally by flow cytrometry. The length of *D *period can be obtained by using flow cytometry data in these equations.

### Bacterial synchronization for initiation of chromosome replication

Synchronization of bacteria for one initiation of chromosome replication was performed as described [30]. Strain CMT934 was grown in glycerol minimal medium at 30°C. At mid log phase, the culture was transferred to 42°C for 1 h to inhibit initiation and complete ongoing rounds of replication. Cells were subsequently shifted to 30°C to allow one initiation event, and after 6 min they were transferred back to 42°C to avoid further initiations. Incorporation of ^3^H-thymidine into TCA-precipitable material showed that this treatment gave rise to one replication round and that it terminated after about 50 min (data not shown).

## Authors' contributions

MASR performed experiments, and helped to design experiments and to draft the manuscript. FM conceived and designed experiments, conducted and analyzed cytometry experiments, and helped to draft the manuscript. AJS conceived the study, its design and coordination, and drafted the manuscript. All authors read and approved the final manuscript.

## References

[B1] Marians KJ Understanding how the replisome works Nat Struct Mol Biol 2008 15 125 127 10.1038/nsmb0208-125 18250630

[B2] Eklund H Uhlin U Färnegardh M Logan DT Nordlund P Structure and function of the radical enzyme ribonucleotide reductase Prog Biophys Mol Biol 2001 77 177 268 10.1016/S0079-6107(01)00014-1 11796141

[B3] Nordlund P Reichard P Ribonucleotide reductases Annu Rev Biochem 2006 75 681 706 10.1146/annurev.biochem.75.103004.142443 16756507

[B4] Mathews CK Sinha NK Are DNA precursors concentrated at replication sites? Proc Natl Acad Sci USA 1982 79 302 306 10.1073/pnas.79.2.302 7043458 PMC345714

[B5] Warner HR Properties of ribonucleoside disphosphate reductase in nucleotide-permeable cells J Bacteriol 1973 115 18 22 4146181 10.1128/jb.115.1.18-22.1973 PMC246205

[B6] Buckstein MH He J Rubin H Characterization of nucleotide pools as a function of physiological state in *Escherichia coli* J Bacteriol 2008 190 718 726 10.1128/JB.01020-07 17965154 PMC2223692

[B7] Reichard P Control of deoxyribonucleotide synthesis in vitro and in vivo Adv Enzyme Reg 1972 10 3 16 10.1016/0065-2571(72)90003-9 4569540

[B8] Reddy GP Mathews CK Functional compartmentation of DNA precursors in T4 phage-infected bacteria J Biol Chem 1978 253 3461 3467 348692

[B9] Butland G Peregrín-Alvarez JM Li J Yang W Yang X Canadien V Starostine A Richards D Beattie B Krogan N Davey M Parkinson J Greenblatt J Emili A Interaction network containing conserved and essential protein complexes in *Escherichia coli* Nature 2005 433 531 537 10.1038/nature03239 15690043

[B10] Chao J Leach M Karam J In vivo functional interaction between DNA polymerase and dCMP-hydroxymethylase of bacteriophage T4 J Virology 1977 24 557 563 562423 10.1128/jvi.24.2.557-563.1977 PMC515967

[B11] Kim J Wheeler LJ Shen R Mathews CK Protein-DNA interactions in the T4 dNTP synthetase complex dependent on gene 32 single-stranded DNA-binding protein Mol Microbiol 2005 55 1502 1514 10.1111/j.1365-2958.2004.04486.x 15720556

[B12] Reichard P Interactions between deoxyribonucleotide and DNA synthesis Ann Rev Biochem 1988 57 349 374 10.1146/annurev.bi.57.070188.002025 3052277

[B13] Mathews CK Slabaugh MB Eukaryotic DNA metabolism: Are deoxyribonucleotides channeled to replication sites? Exp Cell Res 1986 162 285 295 10.1016/0014-4827(86)90335-6 3510878

[B14] Reddy GPV Pardee AP Multienzyme complex for metabolic channeling in mammalian DNA replication Proc Natl Acad Sci USA 1980 77 3312 3316 10.1073/pnas.77.6.3312 6251456 PMC349605

[B15] Guzmán EC Caballero JL Jiménez-Sánchez A Ribonucleoside diphosphate reductase is a component of the replication hyperstructure in *Escherichia coli* Mol Microbiol 2002 43 487 495 10.1046/j.1365-2958.2002.02761.x 11985724

[B16] Fuchs JA Karlstrom HO Warner HR Reichard P Defective gene product in dnaF mutant of *E. coli* Nature 1972 238 69 71 10.1038/238069a0 4558262

[B17] Guarino E Jiménez-Sánchez A Guzmán EC Defective ribonucleoside diphosphate reductase impairs replication fork progression in *Escherichia coli* J Bacteriol 2007 189 3496 3501 10.1128/JB.01632-06 17322311 PMC1855873

[B18] Riola J Guarino E Guzmán EC Jiménez-Sánchez A Differential inhibition of NDP reductase by chemical inactivation and by the thermosensitive mutation *nrdA101 *in *Escherichia coli* Cell Mol Biol Letters 2007 12 70 81 10.2478/s11658-006-0060-0 PMC6275884 17124544

[B19] Norris V Blaauwen T Cabin-Flaman A Doi RH Errington J Harshey R Janniere L Jiménez-Sánchez A Jin DJ Levin PA Mileykovskaya E Minsky A Saier M Skarstad K A Taxonomy of Bacterial Hyperstructures Annu Rev Microbiol 2007 61 309 329 10.1146/annurev.micro.61.081606.103348 17896876

[B20] Norris V Blaauwen T Cabin-Flaman A Doi RH Errington J Harshey R Janniere L Jiménez-Sánchez A Jin DJ Levin PA Mileykovskaya E Minsky A Saier M Skarstad K Functional Taxonomy of Bacterial Hyperstructures Microbiol Mol Biol Rev 2007 71 230 253 10.1128/MMBR.00035-06 17347523 PMC1847379

[B21] Molina F Skarstad K Replication fork and SeqA focus distributions in *Escherichia coli *suggest a replication hyperstructure dependent on nucleotide metabolism Mol Microbiol 2004 52 1597 1612 10.1111/j.1365-2958.2004.04097.x 15186411

[B22] Herrick J Sclavi B Ribonucleotide reductase and the regulation of DNA replication: an old story and an ancient heritage Mol Microbiol 2007 63 22 34 10.1111/j.1365-2958.2006.05493.x 17229208

[B23] Watt RM Wang J Leong M Kung H Cheah KSE Liu D Danchin A Huang JD Visualizing the proteome of *Escherichia coli *an efficient and versatile method for labeling chromosomal coding DNA sequences (CDSs) with fluorescent protein genes Nucleic Acids Res 2007 35 e37 1 11 17272300 10.1093/nar/gkl1158 PMC1874593

[B24] Hiraga S Ichinose C Niki H Yamazoe M Cell cycle-dependent duplication and bidirectional migration of SeqA-associated DNA-protein complexes in *E. coli* Mol Cell 1998 1 381 387 10.1016/S1097-2765(00)80038-6 9660922

[B25] Hiraga S Dynamic localization of bacterial and plasmid chromosomes Ann Rev Genet 2000 34 21 59 10.1146/annurev.genet.34.1.21 11092821

[B26] Molina F Sánchez-Romero MA Jiménez-Sánchez A Dynamic organization of replication forks into factories in *Escherichia coli* Process Biochem 2008 43 1171 1177 10.1016/j.procbio.2008.06.017

[B27] Morigen Odsbu I Skarstad K Growth rate dependent numbers of SeqA structures organize the multiple replication forks in rapidly growing *Escherichia coli* Genes Cells 2009 14 643 657 10.1111/j.1365-2443.2009.01298.x 19371375

[B28] Kubitschek HE Woldringh CL Cell elongation and division probability during the *Escherichia coli *growth cycle J Bacteriol 1983 153 1379 1387 6337997 10.1128/jb.153.3.1379-1387.1983 PMC221788

[B29] Trueba FJ Koppes LJ Exponential growth of *Escherichia coli *B/r during its division cycle is demonstrated by the size distribution in liquid culture Arch Microbiol 1998 169 491 496 10.1007/s002030050601 9575234

[B30] Yamazoe M Adachi S Kanaya S Ohsumi K Hiraga S Sequential binding of SeqA protein to nascent DNA segments at replication forks in synchronized cultures of *Escherichia coli* Mol Microbiol 2005 55 289 298 10.1111/j.1365-2958.2004.04389.x 15612935

[B31] Den Blaauwen T Aarsman MEG Wheeler LJ Nanninga N Pre-replication assembly of *E. coli *replisome components Mol Microbiol 2006 62 695 708 10.1111/j.1365-2958.2006.05417.x 16999830

[B32] Wang X Reyes-Lamothe R Sherratt DJ Visualizing genetic loci and molecular machines in living bacteria Biochem Soc Trans 2008 36 749 753 10.1042/BST0360749 18631152

[B33] Müller-Hill B What is life? The paradigm of DNA and protein cooperation at high local concentrations Mol Microbiol 2006 60 253 255 10.1111/j.1365-2958.2006.05126.x 16573677

[B34] Datsenko KA Wanner BL One-step inactivation of chromosomal genes in *Escherichia coli *K-12 using PCR products Proc Natl Acad Sci USA 2000 97 6640 6645 10.1073/pnas.120163297 10829079 PMC18686

[B35] Uzzau S Figueroa-Bossi N Rubino S Bossi L Epitope tagging of chromosomal genes in *Salmonella* Proc Natl Acad Sci USA 2001 98 15264 15269 10.1073/pnas.261348198 11742086 PMC65018

[B36] Cherepanov PP Wackernagel W Gene disruption in *Escherichia coli*: TcR and KmR cassettes with the option of Flp-catalyzed excision of the antibiotic-resistance determinant Gene 1995 158 9 14 10.1016/0378-1119(95)00193-A 7789817

[B37] Fossum S Soreide S Skarstad K Lack of SeqA focus formation specific DNA binding and proper protein multimerization in the *Escherichia coli *sequestration mutant seqA2 Mol Microbiol 2003 47 619 632 10.1046/j.1365-2958.2003.t01-1-03329.x 12535065

[B38] Powell EO Growth rate and generation time of bacteria, with special reference to continuous culture J Gen Microbiol 1956 15 492 511 13385433 10.1099/00221287-15-3-492

[B39] Wold S Skarstad S Steen HB Stokke T Boye E The initiation mass for DNA replication in *Escherichia coli *K-12 is dependent on growth rate EMBO J 1994 13 2097 2102 8187762 10.1002/j.1460-2075.1994.tb06485.x PMC395061

[B40] Sueoka N Yoshikawa H The chromosome of Bacillus subtilis. Theory of marker frequency analysis Genetics 1965 52 747 757 4953222 10.1093/genetics/52.4.747 PMC1210937

